# The Colorectal cancer RISk Prediction (CRISP) trial: a randomised controlled trial of a decision support tool for risk-stratified colorectal cancer screening

**DOI:** 10.3399/BJGP.2022.0480

**Published:** 2023-04-04

**Authors:** Jon D Emery, Mark A Jenkins, Sibel Saya, Patty Chondros, Jasmeen Oberoi, Shakira Milton, Kitty Novy, Emily Habgood, Napin Karnchanachari, Marie Pirotta, Lyndal Trevena, Adrian Bickerstaffe, Richard De Abreu Lourenço, Anna Crothers, Driss Ait Ouakrim, Louisa Flander, James G Dowty, Fiona M Walter, Malcolm Clark, Sally Doncovio, Dariush Etemadmoghadam, George Fishman, Finlay Macrae, Ingrid Winship, Jennifer G McIntosh

**Affiliations:** Department of General Practice and Centre for Cancer Research, University of Melbourne, Victoria, Australia; The Primary Care Unit, Department of Public Health & Primary Care, University of Cambridge, Cambridge, UK.; Centre for Epidemiology and Biostatistics, Melbourne School of Population and Global Health, University of Melbourne, Victoria, Australia.; Department of General Practice and Centre for Cancer Research, University of Melbourne, Victoria, Australia.; Department of General Practice, University of Melbourne, Victoria, Australia.; Department of General Practice and Centre for Cancer Research, University of Melbourne, Victoria, Australia.; Department of General Practice and Centre for Cancer Research, University of Melbourne, Victoria, Australia.; Department of General Practice and Centre for Cancer Research, University of Melbourne, Victoria, Australia.; Department of General Practice and Centre for Cancer Research, University of Melbourne, Victoria, Australia.; Department of General Practice and Centre for Cancer Research, University of Melbourne, Victoria, Australia.; Department of General Practice, University of Melbourne, Victoria, Australia.; Faculty of Medicine and Health, School of Public Health, University of Sydney, Sydney, Australia.; Centre for Epidemiology and Biostatistics, Melbourne School of Population and Global Health, University of Melbourne, Victoria, Australia.; Centre for Health Economics Research and Evaluation, University of Technology Sydney, Sydney, Australia.; Centre for Health Economics Research and Evaluation, University of Technology Sydney, Sydney, Australia.; Centre for Epidemiology and Biostatistics, Melbourne School of Population and Global Health, University of Melbourne, Victoria, Australia.; Centre for Epidemiology and Biostatistics, Melbourne School of Population and Global Health, University of Melbourne, Victoria, Australia.; Centre for Epidemiology and Biostatistics, Melbourne School of Population and Global Health, University of Melbourne, Victoria, Australia.; Department of General Practice, University of Melbourne, Victoria, Australia; The Primary Care Unit, Department of Public Health & Primary Care, University of Cambridge, Cambridge, UK; Wolfson Institute of Population Health, Queen Mary University of London, London, UK.; IPN Medical Centres, Victoria, Australia.; Prevention and Population Health Branch, Department of Health, Victoria, Australia.; Prevention and Population Health Branch, Department of Health, Victoria, Australia.; Consumer Advisory Group, Primary Care Collaborative Cancer Clinical Trials Group (PC4), Melbourne, Australia.; Department of Medicine, University of Melbourne, Victoria, Australia; Colorectal Medicine and Genetics, Royal Melbourne Hospital, Melbourne, Australia.; Department of Medicine, University of Melbourne, Victoria, Australia; Genomic Medicine, Royal Melbourne Hospital, Melbourne, Australia.; Department of General Practice and Centre for Cancer Research, University of Melbourne, Victoria, Australia; HumaniSE Lab, Department of Software Systems and Cybersecurity, Monash University, Victoria, Australia.

**Keywords:** clinical decision support, colorectal neoplasms, early detection of cancer, general practice, primary care, referral and consultation

## Abstract

**Background:**

A risk-stratified approach to colorectal cancer (CRC) screening could result in a more acceptable balance of benefits and harms, and be more cost-effective.

**Aim:**

To determine the effect of a consultation in general practice using a computerised risk assessment and decision support tool (Colorectal cancer RISk Prediction, CRISP) on risk-appropriate CRC screening.

**Design and setting:**

Randomised controlled trial in 10 general practices in Melbourne, Australia, from May 2017 to May 2018.

**Method:**

Participants were recruited from a consecutive sample of patients aged 50–74 years attending their GP. Intervention consultations included CRC risk assessment using the CRISP tool and discussion of CRC screening recommendations. Control group consultations focused on lifestyle CRC risk factors. The primary outcome was risk-appropriate CRC screening at 12 months.

**Results:**

A total of 734 participants (65.1% of eligible patients) were randomised (369 intervention, 365 control); the primary outcome was determined for 722 (362 intervention, 360 control). There was a 6.5% absolute increase (95% confidence interval [CI] = −0.28 to 13.2) in risk-appropriate screening in the intervention compared with the control group (71.5% versus 65.0%; odds ratio [OR] 1.36, 95% CI = 0.99 to 1.86, *P* = 0.057). In those due CRC screening during follow-up, there was a 20.3% (95% CI = 10.3 to 30.4) increase (intervention 59.8% versus control 38.9%; OR 2.31, 95% CI = 1.51 to 3.53, *P*<0.001) principally by increasing faecal occult blood testing in those at average risk.

**Conclusion:**

A risk assessment and decision support tool increases risk-appropriate CRC screening in those due screening. The CRISP intervention could commence in people in their fifth decade to ensure people start CRC screening at the optimal age with the most cost-effective test.

## BACKGROUND

Australia has one of the highest incidence rates of colorectal cancer (CRC) worldwide.^1^^,^^2^ A range of screening tests can reduce CRC mortality including faecal occult blood testing (FOBT)^3^^,^^4^ and flexible sigmoidoscopy.^5^ A recently reported trial of colonoscopy demonstrated reduced risk of CRC but an uncertain effect on CRC mortality.^6^ An Australian microsimulation model found that biennial immunochemical FOBT (iFOBT) was the most cost-effective approach relative to other tests.^7^

Risk-stratified approaches to CRC screening have been proposed where those at higher CRC risk have more invasive tests and commence screening at a younger age.^8^^,^^9^ The Australian National Health and Medical Research Council (NHMRC) recommends biennial iFOBT screening from age 50–74 years for those at average risk of CRC.^10^ For those at moderately increased risk, iFOBT-based screening is recommended from age 40 years and colonoscopy screening from age 50 years; for individuals at higher familial risk, iFOBT-based screening commences from age 35 years and colonoscopy screening from age 45 years.^11^

**Table:** How this fits in

Using risk models that account for family history, lifestyle, and medical history could tailor colorectal cancer (CRC) screening and determine starting age and screening test. This could be more cost-effective than population screening. This randomised controlled trial found that using the Colorectal cancer RISk Prediction (CRISP) risk tool in general practice can increase risk-appropriate CRC screening in those due screening. Its effect is more uncertain in patients who are up to date with screening. The CRISP intervention could be used in people in their fifth decade to ensure people start CRC screening at the optimal age with the most cost-effective screening test.

There are discrepancies between Australian recommendations and actual screening behaviours. Approximately 18% of people at average risk are being screened by colonoscopy, whereas 64% at moderate risk and 56% at high risk of CRC are receiving no screening at all.^12^ Within the Australian National Bowel Cancer Screening Program (NBCSP) participation rates are only 43.5%.^13^

Internationally, there are guidelines that apply family history criteria for risk-stratified CRC screening, with colonoscopy for those at increased risk,^14^ but family history alone is a poor discriminator of CRC risk.^15^ Risk-prediction models exist that incorporate multiple risk factors and have better discrimination.^16^ To translate these models into practice requires risk assessment tools to tailor CRC screening,^17^ but whether such tools offer a cost-effective approach to implement risk-stratified screening is uncertain.^18^

The Colorectal cancer RISk Prediction (CRISP) trial aimed to test the effect of a health consultation in Australian general practice using a risk assessment and decision support tool (the CRISP tool) on risk-appropriate CRC screening.

## METHOD

The trial protocol has been published elsewhere.^19^ The trial design was a stratified randomised controlled trial in 10 general practices in Melbourne, Australia, with patient randomisation (Australian and New Zealand Clinical Trial Registry reference number: ACTRN12616001573448).

### Participants

Eligible participants were aged 50–74 years and able to comprehend written English and give informed consent. Exclusion criteria were: previous diagnosis of CRC or inflammatory bowel disease; current rectal bleeding; and known genetic predisposition to CRC.

Patients aged 50–74 years attending a GP were recruited consecutively from the waiting room and taken into a private room to confirm eligibility and obtain informed consent. An online baseline questionnaire was completed before randomisation.

### Intervention group

The intervention occurred before the participant’s consultation with their GP and involved a standardised consultation delivered by a research assistant in which the participant’s risk of CRC was assessed using the CRISP tool; risk-appropriate CRC screening recommendations were discussed and a report provided to the participant and their GP. This was designed to model the role of a practice nurse, the most likely method of implementation of the CRISP tool in general practice, based on this current study group’s previous developmental studies.^20^

The CRISP tool is a web-based application that calculates an individual’s 5-year and lifetime risk of developing CRC (http://crisp.org.au/crisp-clinic) and recommends CRC screening (Supplementary Appendix S1).

Participants were encouraged to discuss the CRISP report with their GP. Those due an iFOBT test were shown how to complete the test and their GP was expected to order an iFOBT. Participants due iFOBT screening received an SMS (short message service) reminder at 1 month to complete the test. If the participant reported a history of polyps, the GP received a summary sheet about NHMRC polyp surveillance guidelines, asking them to arrange colonoscopic surveillance. These components were part of a complex intervention to improve risk-appropriate CRC screening.^21^

### Control group

Those randomised to the control group were directed to an online presentation of the Cancer Council Victoria ‘Cut your Cancer Risk’ brochure. The research assistant discussed the information using a standardised script; the focus was on modifiable lifestyle factors to reduce cancer risk. Participants received a copy of the brochure and continued with usual care.

### Outcomes and measures

The primary outcome was the proportion of participants who had completed risk-appropriate CRC screening at 12-month follow-up. In the intervention group, the risk category was defined using the CRISP-calculated 5-year CRC risk; for the control group, it was determined by their family history in accordance with the NHMRC-endorsed guidelines that were current at the time of recruitment (Supplementary Appendix S1).^10^^,^^22^ Appropriateness of screening for both groups was determined by an assessment of previous screening and concordance with the recommended mode and frequency of screening for each risk group (Supplementary Appendix S1).

CRC screening was obtained from multiple sources: self-report; GP record audit; Medicare Benefits Schedule (MBS, see Supplementary Table S1); the NBCSP; and the Victorian Admitted Episodes Dataset (VAED). In the current study self-reported data were used only where objective data from these clinical and administrative sources were unavailable.

A clinical subcommittee reviewed blinded screening data for cases where there were discrepancies between data sources or to review participants with complex polyp histories (Supplementary Appendix S1).

Additional measures included: demographics and clinical variables at baseline, and the following secondary outcomes:

- risk perception, absolute, and comparative risk;^23^^,^^24^
- State–Trait Anxiety Inventory (STAI) scale;^25^
- cancer-specific anxiety;^26^^,^^27^
- intentions to have CRC screening;
- clinical outcomes of screening tests (to be reported with 5-year follow-up); and
- healthcare service utilisation and costs related to CRC screening at 12 months obtained from GP records, MBS, VAED, and NBCSP data (Supplementary Appendix S1).

Participant-completed measures (a–d) were collected at baseline, 1, 6, and 12 months post-randomisation.

### Sample size

The original sample size was 278 participants per group, based on historic estimates of risk-appropriate screening of <5%.^28^ The trial steering committee met in February 2018 and reviewed blinded self-reported CRC screening in 397 participants, suggesting that risk-appropriate screening at baseline was as much as 25%. The committee recommended an increase in the sample size to 366 per group. Allowing for 10% attrition over 12 months, this gave at least 80% power with a two-sided 5% level of significance to detect a minimum 10% difference in the proportion who were risk-appropriately screened, assuming 25% of control participants received risk-appropriate screening at 12 months.

### Randomisation and masking

Participants were automatically randomised after the baseline questionnaire. The random allocation sequence, stratified by general practice, was computer-generated by the trial statistician with a 1:1 allocation ratio using random permuted block sizes of four, six, and eight within each stratum. This randomisation sequence was incorporated into the online platform used to collect baseline data and redirect the browser to the CRISP tool or the control presentation. Participants were told the trial was about bowel cancer prevention and therefore blinded to allocation.

### Blinding

For telephone follow-up of non-responders, and extraction and analysis of health service utilisation data, research staff were blinded to group assignment. All statistical analyses were performed blinded to group assignment.

### Statistical methods

All randomised participants who did not withdraw their data were included in the primary analysis. Those who died before the 12 months’ follow-up were excluded for the primary outcome, but their survey responses for secondary outcomes are included in the study. For the primary outcome, logistic regression was used to estimate the odds ratio (OR). A generalised linear model was used with the identity link function and binomial family to estimate the absolute difference in the proportion of risk-appropriate screening between groups. All regression models included the randomisation stratification factor of general practice as a fixed effect. The absolute (between-group difference in the proportions) and relative (OR) estimated effect sizes are presented with their respective 95% confidence interval (CI), and the *P*-value estimated using logistic regression.

Comparisons between groups on continuous secondary outcomes used a linear mixed-effects model that included trial group, general practice, and time (baseline, 1, 6, and 12 months) as fixed effects and individuals as random effects, with two-way interactions between group and time, except for baseline where study group means were constrained to be equal. Comparisons between groups on binary secondary endpoints with repeated outcome measures were performed using logistic regression, using generalised estimating equations with robust standard errors, with general practice as the covariate.

Based on review of the blinded data, the trial steering committee agreed to conduct an explanatory analysis using a statistical test for interaction to examine if the intervention effect was modified by whether participants were due CRC screening during follow-up.^29^ To assess for potential contamination, the number of iFOBTs ordered by GPs at 2 and 4 weeks after recruitment was examined. Planned sensitivity analyses are described in Supplementary Appendix S2. Analyses were conducted in Stata (version 15).

Costs for delivering the intervention and associated with screening utilisation were expressed as the mean expenditure and associated 95% CI for iFOBT and colonoscopy over the period of the trial for each group, including overscreening. The cost per appropriately screened individual was calculated for the CRISP intervention compared with usual care based on the primary outcome measure and for those due screening at baseline.

## RESULTS

**Figure 1.**
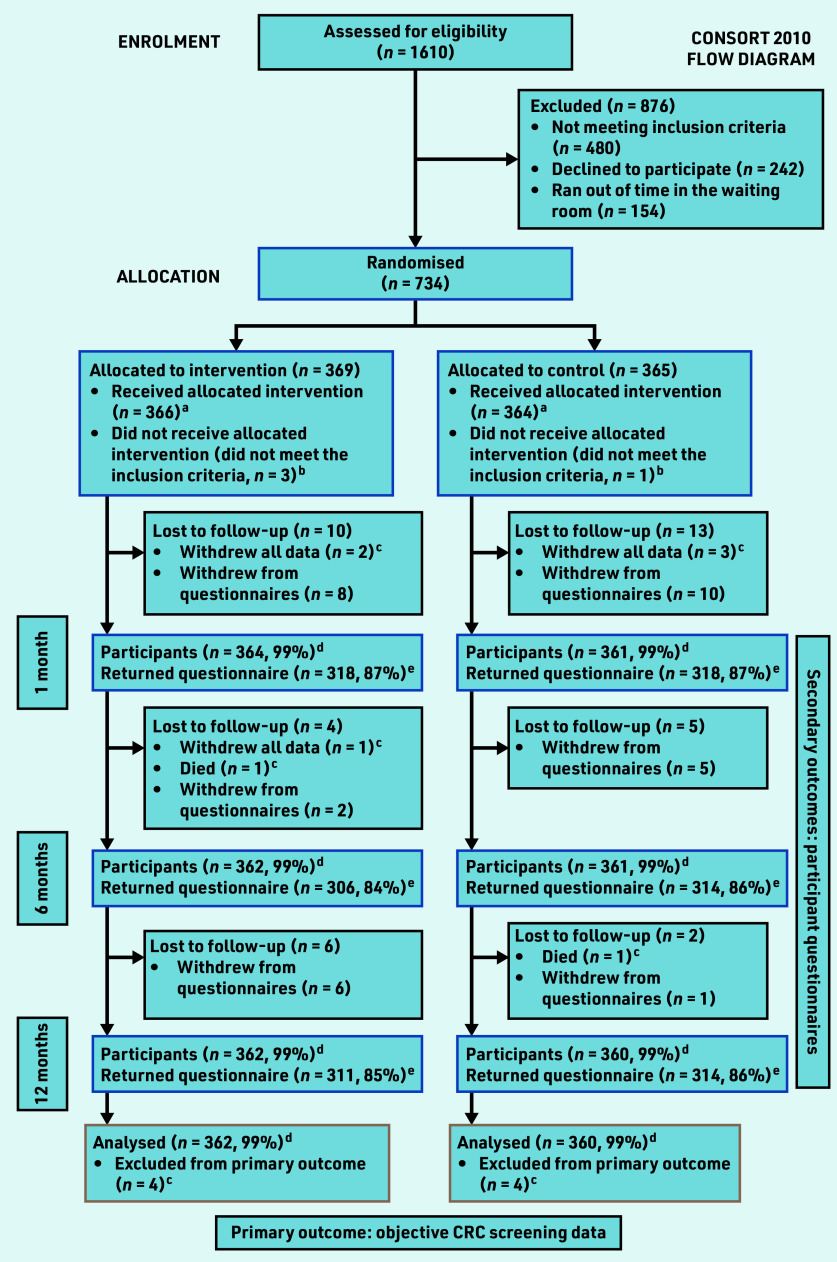
*Consort diagram showing number of participants assessed for eligibility, randomised, and available for analysis of primary and secondary outcomes. ^a^ Denominator for percentages is those who received the allocated intervention in each group (366 for intervention group, 364 for control group). ^b^Participants who did not meet the inclusion criteria were excluded from all analyses. ^c^Participants who withdrew all data or who died before the primary endpoint were excluded from all analyses (four in each group). ^d^The number of participants remaining for analysis of the primary outcome at each timepoint. ^e^The number of participants who returned questionnaires at each timepoint contributing data to the analysis of secondary outcomes.*

Between 9 May 2017 and 4 May 2018, 1610 patients were approached of whom 1128 were eligible. In total, 734 (65.1%) consented and were randomised (Figure 1). Three participants in each trial group withdrew all data, and one participant in each group died during 12 months’ follow-up. Four participants were identified as ineligible post-randomisation (*n* = 3 intervention, *n* = 1 control group). One participant in each group died during the 12 months’ follow-up. All participants received the allocated interventions as intended. Age and sex were similar between those recruited and those who declined to participate (Supplementary Table S2). Participant characteristics were balanced between groups (Table 1). The distribution of socioeconomic advantage of the trial cohort was comparable with the population of Melbourne.^30^ The majority of participants (95%–96%) were in an average CRC risk category based on the CRISP risk prediction model or NHMRC criteria (Table 1).^10^^,^^22^

**Table 1.** Characteristics by trial group (*N* = 724)^a^

**Characteristic**	**Intervention group (*n* = 363)^b^**	**Control group (*n* = 361)^b^**
**Age, years, mean (SD)**	63.28 (6.83)	63.09 (6.76)

**Sex**		
Female	218 (60.1)	215 (59.6)
Male	145 (39.9)	146 (40.4)

**Born in Australia**	250 (68.9)	246 (68.1)

**Index of Relative Socioeconomic Advantage and Disadvantage (deciles)^c^ for participants residence**		
1–3	47 (12.9)	47 (13.0)
4–7	67 (18.5)	67 (18.6)
8–10	249 (68.6)	247 (68.4)

**English spoken at home**	336 (92.6)	336 (93.1)

**Current relationship status**		
Single	54 (14.9)	61 (16.9)
In a relationship	23 (6.3)	35 (9.7)
Married	218 (60.1)	209 (57.9)
Separated/divorced	38 (10.5)	32 (8.9)
Widowed	30 (8.3)	24 (6.6)

**Highest level of education completed**		
Never completed high school	80 (22.0)	59 (16.3)
Completed high school only	84 (23.1)	78 (21.6)
TAFE qualification or similar	55 (15.2)	81 (22.4)
University degree or similar	144 (39.7)	143 (39.6)

**Risk category based on NHMRC family history criteria (2005)**		
Average	347 (95.6)	342 (94.7)
Moderate	11 (3.0)	15 (4.2)
High	5 (1.4)	4 (1.1)

**Risk category based on NHMRC family history criteria (2017)^d^**		
Average	348 (96.4)	340 (95.0)
Moderate	11 (3.0)	15 (4.2)
High	2 (0.6)	3 (0.8)

**Risk category based on CRISP model**		
Average	342 (94.2)	N/A
Moderate	16 (4.4)	
High	5 (1.4)	

a *One person in each arm died before the primary endpoint (12 months) so were excluded from the primary outcome. Their survey responses are included for the secondary outcomes.*

b *Data are presented as* n *(%) unless otherwise stated.*

c *Decile 1 is the most disadvantaged and 10 is the most advantaged decile.*

d *Two participants in the intervention group and three in the control group could not have their family history category determined for the 2017 guidelines because of incomplete data; their family history meant that they were at least moderate risk. CRISP = Colorectal cancer RISk Prediction. NHMRC = National Health and Medical Research Council. N/A = not applicable. TAFE = Technical and Further Education.*

**Figure 2.**
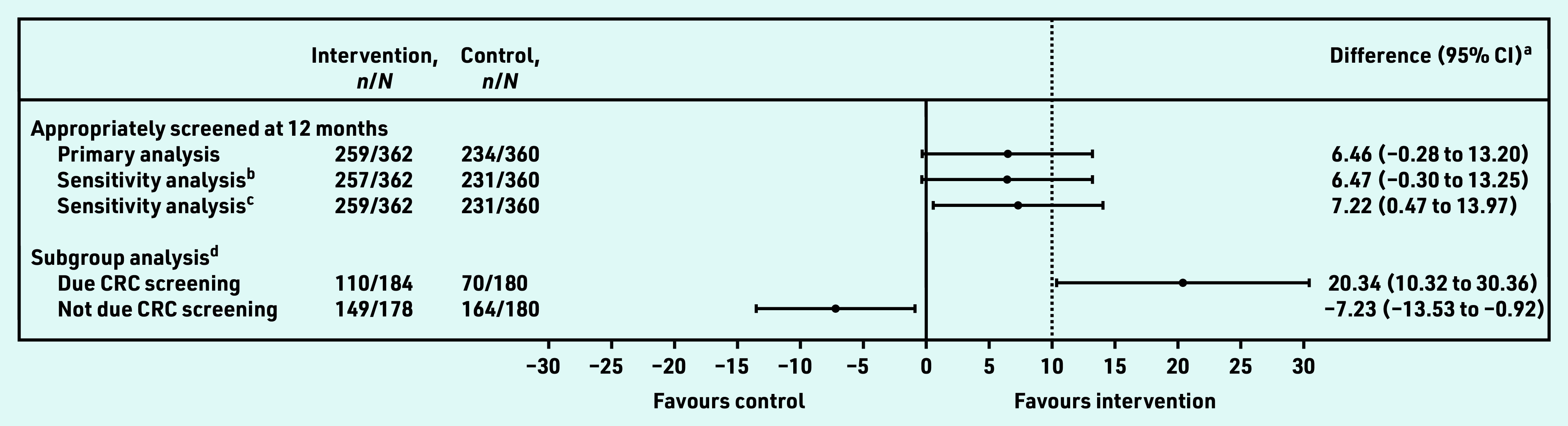
*Forest plot showing estimated effect sizes including planned sensitivity and subgroup analyses. ^a^ Difference in the percentage with appropriate CRC screening at 12-month follow-up between the intervention and control groups and respective 95% CI estimated using generalised linear model with the identity link function and binomial family adjusted for the general practice. ^b^Accounting for data on CRC screening at baseline available to the GP to determine the type of CRC screening that was due. ^c^Excluding three participants in control group with self-reported outcomes only. ^d^Effect modification by whether participants were due CRC screening during 12-month follow-up (*n *= 184 in the intervention group and* n *= 180 in the control group) or not (*n *= 178 in the intervention group and* n *= 180 in the control group); interaction term estimated for between-group difference on the percentage scale 27.6% (95% CI = 15.7 to 39.5),* P*-value for effect modification <0.001. CRC = colorectal cancer.*

Objective CRC screening information was ascertained for 99.6% (*n* = 722/725) participants; three control participants had self-reported data only. Of these 722 participants, 71.5% (*n* = 259/362) had risk-appropriate screening in the intervention group compared with 65.0% (*n* = 234/360) in the control group, a 6.5% absolute increase between groups. The authors are 95% confident that the true between-group difference lies between −0.28% and 13.2%, which includes the hypothesised minimally important value of 10% (Table 2 and Figure 2). Estimates adjusted for risk group remained relatively unchanged (data not shown). The sensitivity analyses demonstrated similar patterns to the primary analysis (Figure 2).

**Table 2.** Appropriate colorectal cancer screening at 12-month follow-up between trial groups (*N* = 722)^a^

**Appropriately screened at 12 months**	**Intervention group (*n* = 362)**		**Control group (*n* = 360)**		**Difference, % (95% CI)^b^**	**OR (95% CI)^c^**	***P*-value^c^**

	***n***	**%**	***n***	**%**			
**Primary analysis**	259	71.5	234	65.0	6.46 (−0.28 to 13.20)	1.36 (0.99 to 1.86)	0.057

**Sensitivity analysis^d^**	257	71.0	231	64.2	6.47 (−0.30 to 13.25)	1.37 (1.00 to 1.88)	0.048

**Sensitivity analysis^e^**	259	71.5	231	64.2	7.22 (0.47 to 13.97)	1.41 (1.03 to 1.93)	0.033

**Subgroup analysis^f^**							
Due CRC screening	110	59.8	70	38.9	20.34 (10.32 to 30.36)	2.31 (1.51 to 3.53)	<0.001
Not due CRC screening	149	83.7	164	91.1	−7.23 (−13.53 to −0.92)	0.51 (0.26 to 0.97)	0.042

a *One person from each trial group was excluded because they died before the 12 months of follow-up.*

b *Difference in the percentage and respective 95% CI between the intervention and control groups estimated using generalised linear model with the identity link function and binomial family adjusted for general practice.*

c *OR, respective 95% CI, and* P*-value estimated using logistic regression adjusted for general practice.*

d *Accounting for data on CRC screening at baseline available to the GP to determine the type of CRC screening that was due.*

e *Excluding three participants in control group with self-reported outcomes only.*

f *Effect modification by whether participants were due CRC screening during 12-month follow-up (*n *= 184 in the intervention group and* n *= 180 in the control group) or not (*n *= 178 in the intervention group and* n *= 180 in the control group); interaction term estimated for between-group difference on the percentage scale 27.6% (95% CI = 15.7 to 39.5),* P*-value for effect modification <0.001. CRC = colorectal cancer. OR = odds ratio.*

In the intervention group, 50.8% (*n* = 184/362) of participants were due CRC screening at baseline or within the next 12 months of follow-up, and 50.0% (*n* = 180/360) in the control group. There was strong evidence for effect modification (*P*<0.001); of those who were due CRC screening, 59.8% (*n* = 110/184) in the intervention group compared with 38.9% (*n* = 70/180) in the control group had risk-appropriate screening at 12 months (estimated absolute group-difference in proportions: 20.3%, 95% CI = 10.3 to 30.4, *P*<0.001). In those who were not due screening, the intervention was associated with reduced risk-appropriate screening at 12 months (intervention group 83.7%, *n* = 149/178 versus control group 91.1%, *n* = 164/180, estimated absolute group-difference in proportions −7.2% [95% CI = −13.5 to −0.9, *P* = 0.042]) (Table 2 and Figure 2).

More participants were overscreened in the intervention group compared with the control group. Thirty-six intervention group participants (9.9%) were overscreened at 12 months (*n* = 17 iFOBT, 47.2% and *n* = 19 colonoscopies, 52.8%), compared with 18 (5.0%) in the control group (*n* = 6 iFOBT, 33.3% and *n* = 12 colonoscopies, 66.7%) (data not shown). GPs ordered an iFOBT in 11.6% and 14.6% of intervention participants at 2 and 4 weeks, respectively, compared with 0.3% and 1.1% of control participants, demonstrating minimal contamination between groups. These process data were consistent with the intervention acting mainly through GPs ordering more iFOBT tests in those at average risk of CRC (data not shown).

At 1-month follow-up those in the intervention group were more likely than the control group to intend completing an iFOBT in the next 3 months (27.0% versus 14.8%, OR 2.16, 95% CI = 1.46 to 3.21, *P*<0.001) (Table 3). There were no observed effects on participants’ intentions to have a colonoscopy or modify their lifestyle. There were no differences between groups at any timepoint on general or cancer-specific anxiety or absolute risk perception (Table 4).

**Table 3.** Intentions and self-reported behaviours to manage risk of colorectal cancer between trial groups (*N* = 724)^a^

**Intention/behaviour**	**Intervention group (*n* = 363)**		**Control group (*n* = 361)**		**OR**	**95% CI^b^**	***P*-value**

	***n***	**%**	***n***	**%**			
**In the 3 months, I intend to:**							
Look for further information^c^							
1 month	35	11.0	33	10.4	1.08	0.65 to 1.78	0.769
6 months	35	11.4	24	7.7	1.52	0.89 to 2.61	0.129
12 months	36	11.6	29	9.2	1.28	0.76 to 2.15	0.353
Consult with a health professional about my cancer risk^c^							
1 month	36	11.3	26	8.2	1.43	0.84 to 2.45	0.190
6 months	36	11.8	25	8.0	1.51	0.89 to 2.58	0.130
12 months	32	10.3	33	10.5	0.98	0.58 to 1.65	0.945
Complete a bowel cancer screening test using FOBT^c^							
1 month	86	27.0	47	14.8	2.16	1.46 to 3.21	<0.001
6 months	75	24.5	62	19.8	1.32	0.91 to 1.93	0.148
12 months	75	24.1	57	18.2	1.43	0.97 to 2.10	0.069
Have a colonoscopy to screen for bowel cancer							
1 month	22	6.9	23	7.2	0.91	0.49 to 1.68	0.759
6 months	25	8.2	19	6.1	1.39	0.74 to 2.61	0.306
12 months	26	8.4	21	6.7	1.30	0.70 to 2.40	0.402
Make changes to my diet or eating habits							
1 month	60	18.9	66	20.8	0.88	0.59 to 1.29	0.506
6 months	68	22.2	75	23.9	0.92	0.63 to 1.34	0.663
12 months	79	25.4	76	24.2	1.05	0.73 to 1.52	0.775
Make changes to my physical activity or exercise							
1 month	103	32.4	95	29.9	1.15	0.82 to 1.61	0.416
6 months	117	38.2	123	39.2	0.96	0.69 to 1.33	0.804
12 months	139	44.7	123	39.2	1.25	0.91 to 1.72	0.170
Ask my GP for a referral to a specialist							
1 month	11	3.5	14	4.4	0.75	0.34 to 1.68	0.485
6 months	16	5.2	10	3.2	1.68	0.74 to 3.79	0.211
12 months	15	4.8	14	4.5	1.10	0.51 to 2.35	0.815

**In the last month, I have:**							
Looked for further information about bowel cancer^d^							
1 month	23	7.4	15	4.7	1.60	0.81 to 3.14	0.176
6 months	23	7.5	31	9.9	0.73	0.41 to 1.28	0.269
12 months	49	15.8	39	12.4	1.36	0.86 to 2.15	0.186
Consulted with a health professional about my cancer risk							
1 month	45	14.2	29	9.1	1.60	0.98 to 2.63	0.063
6 months	41	13.4	36	11.5	1.14	0.71 to 1.85	0.583
12 months	57	18.3	45	14.3	1.38	0.90 to 2.10	0.142
Found out about further test for bowel cancer							
1 month	36	11.3	26	8.2	1.42	0.83 to 2.42	0.197
6 months	24	7.8	26	8.3	0.93	0.52 to 1.67	0.816
12 months	51	16.4	35	11.1	1.56	0.98 to 2.47	0.061
Made changes to my diet or eating habits							
1 month	53	16.7	58	18.2	0.90	0.60 to 1.36	0.629
6 months	90	29.4	97	30.9	0.97	0.69 to 1.37	0.864
12 months	108	34.7	105	33.4	1.08	0.78 to 1.50	0.653
Asked my GP for a referral to a specialist							
1 month	23	7.2	15	4.7	1.44	0.75 to 2.79	0.276
6 months	26	8.5	22	7.0	1.19	0.65 to 2.17	0.578
12 months	41	13.2	30	9.6	1.46	0.88 to 2.42	0.143
Been referred to a specialist familial cancer clinic to discuss my family history of cancer							
1 month	4	1.3	4	1.3	1.02	0.26 to 4.11	0.973
6 months	5	1.6	7	2.2	0.73	0.23 to 2.32	0.591
12 months	8	2.6	4	1.3	2.06	0.61 to 6.93	0.245
Attended a familial cancer clinic to discuss my family history of cancer							
1 month	3	0.9	2	0.6	1.57	0.27 to 9.21	0.616
6 months	2	0.7	4	1.3	0.51	0.09 to 2.88	0.448
12 months	5	1.6	5	1.6	1.01	0.28 to 3.64	0.982

a *Total sample: 318 in intervention group and 318 in control group at 1 month; 306 in intervention group and 314 in control group at 6 months; and 311 in intervention group and 314 in control group at 12 months.*

b *OR with respective 95% CI estimated using logistic regression using generalised estimating equations with robust standard errors, trial group, time (baseline, 1, 6, and 12 months), risk group, and general practice as fixed effects, with two-way interactions between trial group and time. Estimates not adjusted for risk group were similar (data not shown).*

c *For this item at 6 months there was one additional person in the control group who had a missing response (n = 313).*

d *For this item sample size at 1 month was 312 in intervention group and 316 in control group. FOBT = faecal occult blood testing. OR = odds ratio.*

**Table 4.** General and cancer-specific anxiety and risk perception between trial groups (*N* = 724)^a^

**Characteristic**	**Intervention group (*n* = 363)**		**Control group (*n* = 361)**		**Difference**	**95% CI^b^**	***P*-value**

	**Mean**	**SD**	**Mean**	**SD**			
**Generalised anxiety (STAI)^c^**							
Baseline	9.05	−3.68	9.17	−3.55	—		
1 month	9.68	−3.73	9.77	−3.69	−0.01	−0.55 to 0.54	0.983
6 months	10.07	−4.10	9.77	−3.67	0.34	−0.24 to 0.91	0.248
12 months	9.60	−3.44	10.11	−4.14	−0.53	−1.11 to 0.04	0.068

**Cancer-specific anxiety**							
Baseline	6.94	−1.57	6.95	−1.51	—		
1 month	7.27	−1.68	7.20	−1.85	0.06	−0.18 to 0.31	0.608
6 months	7.22	−1.79	7.16	−1.58	0.09	−0.15 to 0.32	0.477
12 months	7.31	−2.01	7.27	−1.78	0.06	−0.21 to 0.33	0.670

**Mean perceived risk of colorectal cancer (0%–100%)^d^**							
Baseline	19.33	−19.36	21.98	−20.45	—		
1 month	22.86	−19.95	25.79	−20.73	−1.59	−4.41 to 1.22	0.267
6 months	24.56	−20.39	25.80	−19.75	−0.25	−3.05 to 2.55	0.859
12 months	28.20	−21.59	27.78	−20.38	1.41	−1.49 to 4.32	0.339

a *Total sample for mean and SD: 318 in intervention group and 318 in control group at 1 month; 306 in intervention group and 315 in control group at 6 months; 311 in intervention group and 315 in control group at 12 months.*

b *Mean in the intervention minus the mean in the control arm with respective 95% CI estimated using linear mixed-effects model that included trial group, risk group, general practice, and time (baseline, 1, 6, and 12 months) as fixed effects and individuals treated as random effects, with two-way interactions between trial group and time, except for baseline where trial group means were constrained to be equal. Estimates not adjusted for risk group were similar (data not shown).*

c *At 1 month one additional person in the intervention group had a missing response (*n *= 317); at 6 months two additional people in the control group had a missing response (*n *= 313); and at 12 months an additional person in the intervention arm had a missing response (*n *= 310).*

d *At 6 months one additional person in the control group had a missing response (*n *= 314). SD = standard deviation. STAI = State–Trait Anxiety Inventory.*

The total average incremental cost per participant was Australian $223 (Supplementary Table S3). Based on the primary outcome including all participants, this resulted in an average cost per appropriately screened participant of $3436. This effect ranged from being dominated (costlier and resulting in fewer screened individuals) to an incremental cost per appropriately screened individual of $1718 using the 95% CIs of the efficacy endpoint. When analysis was restricted to those individuals due screening at baseline, the cost per appropriately screened individual was $1990 ($1326 to $3979 using the 95% CIs of the efficacy endpoint) (Supplementary Table S4).

## DISCUSSION

### Summary

Using a risk assessment and decision support tool in patients attending general practice increased risk-appropriate CRC screening by 6.5% in the whole intervention cohort. Although the 95% CI includes a true effect size of no difference, the authors cannot preclude a clinically important true intervention effect since the CI includes the possibility of a 13% increase in risk-appropriate screening, higher than originally hypothesised. In an explanatory analysis, the intervention effect was more evident in people who were due CRC screening, with a 20% absolute increase in risk-appropriate screening over 12 months compared with the control group.

### Strengths and limitations

The intended sample size was recruited, with a high accrual rate; participants were representative of the local population. A hierarchical approach was applied to define the primary outcome using objective health services data in preference to self-report, and self-reported information was relied on for only three participants for the primary analysis. To maintain blinding, risk-appropriate screening was defined based on the risk assessment method specific to each trial group. There were complete data for the primary outcome for 99% of trial participants. The sensitivity analyses showed the findings were robust to different assumptions.

Including participants who were not due CRC screening during follow-up diluted the observable effect of the intervention. They were included because in the current study the authors were interested in effects on risk-appropriate screening, both under- and overscreening, in average and increased risk groups. The rate of risk-appropriate screening in the control group who were due screening was only 39%, similar to rates of participation in the national screening programme. It was not possible to confirm whether someone was genuinely due screening in the 12-month follow-up until the authors obtained the objective screening data from health records and knew their baseline risk. The preliminary estimates of baseline risk-appropriate screening, on which the original and revised sample sizes were based, could not adequately account in advance that only 50% of the sample were due CRC screening during the follow-up period. Although guidelines recommend CRC screening in those at increased risk from age 40 years, a decision was made to recruit a sample aged 50–74 years. If a younger cohort had been recruited, an even larger proportion of participants who were not due screening during follow-up would have been included in the study.

### Comparison with existing literature

A previous systematic review of cancer risk assessment tools, by the same study group, found that tools increase intentions to screen but the effects on risk-appropriate screening were unclear.^31^ The current study’s results provide new evidence that this type of intervention could increase risk-appropriate screening, especially in those who are due screening. The systematic review also highlighted methodological limitations in previous trials including cluster randomised designs and low recruitment rates.^31^ The current trial had a high recruitment rate with a broad range of educational and sociodemographic backgrounds, making the results in the current study more generalisable. Randomisation was at the patient level and demonstrated no evidence of contamination; this design minimised selection bias between groups and made it possible to obtain patient-reported outcomes.^32^

Two trials of CRC risk assessment tools have reported^33^^,^^34^ since the systematic review.^31^ One showed no effect on CRC screening;^33^ the other trialled a self-completed tool that resulted in a threefold relative increase in CRC screening.^34^ The current study tested a tool for application in a consultation, based on clinician feedback recommending it be used by practice nurses.^20^ In a study of a self-completed version of the CRISP tool, people who were older, less educated, or who spoke English as a second language found the tool difficult to complete.^35^

The current complex intervention was more than a computerised risk assessment or a simple reminder to complete screening of any kind. It included a discussion about bowel cancer, demonstration of the iFOBT kit, and prompting of GPs to order the risk-appropriate screening test. The ‘attention control’ was designed to account for the non-specific effects of the intervention. This was an efficacy trial to test whether delivery of the CRISP intervention in a standardised way could improve risk-appropriate screening.^36^ The authors of the current study conducted a parallel implementation study of the CRISP intervention in which practice nurses used the CRISP tool, taking between 5 and 10 minutes to conduct the risk assessment.^37^ The current authors recognise that further implementation-effectiveness research is required to understand whether similar results would be achieved if delivered in routine care.

### Implications for practice

The intervention led to higher rates of overscreening in those who were not due screening, mainly through overordering iFOBT tests. With the implementation of the National Cancer Screening Register, it should be possible to reduce this overscreening by determining when someone is due their next iFOBT. This information in the National Cancer Screening Register could also be used to determine when a CRISP risk assessment should be performed. The current intervention aimed to reduce colonoscopies in people at average risk of CRC. Five-year follow-up data will be reported on potential reductions in colonoscopies in average-risk patients and the longer-term cost-effectiveness of the CRISP tool.

Risk-stratified screening targets more intensive screening to populations with higher rates of cancer, and, if fully implemented, would reduce screening intensity in those at lower risk.^38^ Modelling studies show this is cost-effective,^12^^,^^39^ and there are calls to move away from population-based to risk-based approaches to CRC screening.^8^ The incidence of CRC in people aged <50 years is rising because of risk factors such as obesity, smoking, low physical activity, and diet.^40^ This has led to changes in US guidelines to commence CRC screening from age 45 years.^41^ Risk-based screening that accounts for these risk factors, as well as family history, would require implementation of tools such as CRISP.^17^ However, there remain substantial implementation challenges for countries with CRC screening programmes that mail kits to people based on age, such as in Australia and the UK. A risk-based approach would require greater engagement with primary care and integration with the NBCSP to identify those in their 40s at increased risk of CRC. Nearly 90% of Australians aged 45–54 years attend a GP each year,^42^ and most several times, creating opportunities to assess cancer risk. The CRISP intervention could commence in people in their fifth decade as part of a risk-based screening programme to ensure people start CRC screening at the optimal age and with the most cost-effective screening test.

## Data

Data are not available at present.
